# Free flaps monitoring using implantable doppler: our experience and of the literature

**DOI:** 10.1016/j.jpra.2025.08.023

**Published:** 2025-09-05

**Authors:** Roberto Baraziol, Annachiara Tellarini, Diego Faccio, Alice Alban, Ferruccio Paganini, Luigi Valdatta, Federico Tamborini

**Affiliations:** a Department of Plastic Surgery, ASST Spedali Civili di Brescia, Piazzale Spedali Civili, 1, Brescia 25123, Italy; b Division of Plastic and Reconstructive Surgery, Department of Biotechnology and Life Sciences, University of Insubria, Ospedale di Circolo e Fondazione Macchi, Viale Borri, 57, Varese 2100, Italy; c Microsurgery and Hand Surgery Unit, ASST Settelaghi Varese, Ospedale di Circolo e Fondazione Macchi, Viale Borri, 57, Varese 2100, Italy

**Keywords:** Cook swartz, Synovis, Flow coupler, Doppler, Flap, Monitoring, Anastomosis, Patency

## Abstract

**Background:**

Buried free flaps monitoring cannot rely on clinical evaluation, which remains the gold standard in flaps with a skin paddle. A reliable way to monitor buried free flaps is through implantable dopplers, which provide a continuous and direct signal of anastomotic flow. Since the only way to ascertain a suspicion of vascular impairment in buried free flaps is through reoperative exploration, a high specificity is required together with a high sensitivity to avoid unnecessary theatre take-backs

**Methods:**

A systematic literature search was performed to screen three different databases (PubMed, Web of Sciences, and Embase) and using the following keywords: “Cook Swartz” AND “doppler” AND “flap” AND “monitoring” and “Synovis” AND “Flow Coupler” AND “flap” AND “monitoring”. The indicators of efficacy and effectiveness of the two available implantable dopplers were analyzed and compared to our casistics.

**Results:**

The search using Cook Swartz thesaurus identified 116 articles while that of Synovis Flow Coupler 25 articles, of which only 26 and 6, respectively, fully satisfied our inclusion criteria.

**Conclusion:**

The literature search seems to confirm our current practice, with Cook Swartz (Cook Medical, Bloomington, IN) arterial placement preferred for head and neck buried free flaps and Synovis Flow Coupler (Synovis Life Technologies, Inc., St. Paul, MN) for buried DIEP flaps. This preferential placement reflects the higher rate of false positives when a vein is monitored in the head and neck, which is high mobile and prone to respiratory oscillations leading to probe dislocation or missing signal.

## Introduction

Many techniques for flap monitoring following free tissue transfer have been described in addition to clinical monitoring, which is considered the gold standard, with a very low rate of false positive and false negative in experienced hands. However, there is little evidence that any of these techniques is superior to others'.

## Material and methods

Aim of this study was to critically interpret the results of our experience with buried free flaps monitoring using implantable dopplers. To do so, we have made a systematic review of the pertinent literature.

**Figure 1:**
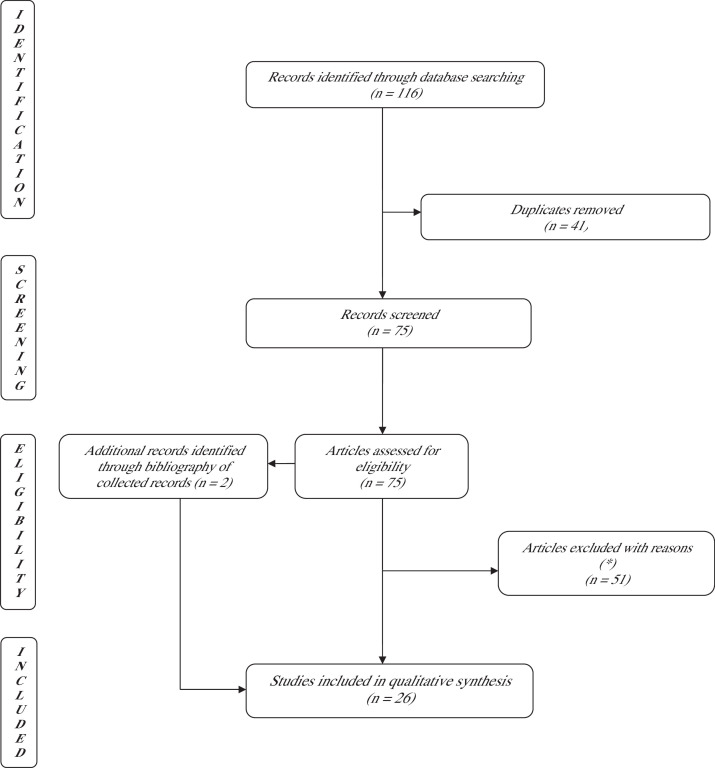
PRISMA flowchart using the keywords: ([{cook Swartz} and {doppler}] and [flap]) and (monitoring). A total of 116 studies were identified through the database search: 26 articles were selected and included.*Review articles, case report, descriptive articles, articles not focusing on test performance.

**Figure 2:**
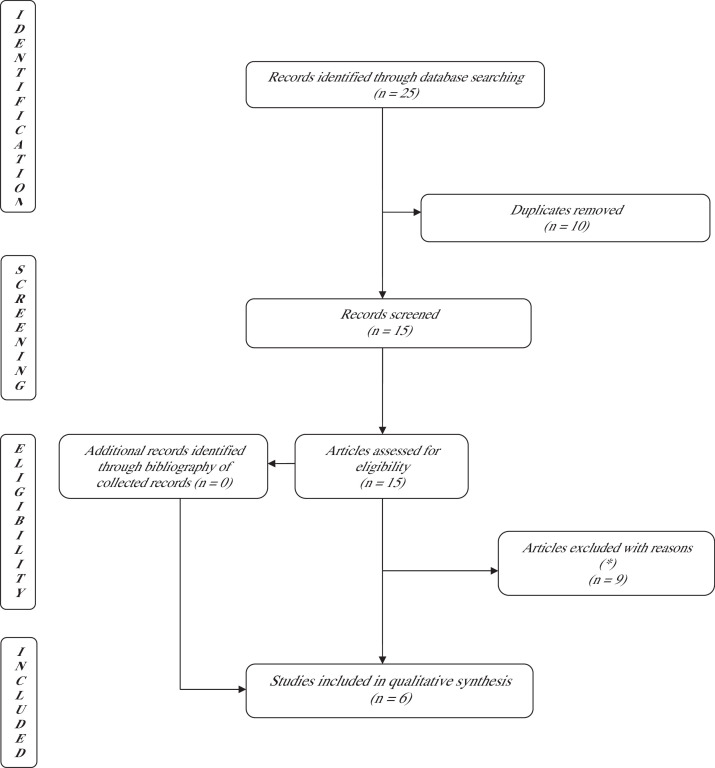
PRISMA flowchart using the keywords: ([{Synovis} and {flow coupler}] and [flap]) and (monitoring). A total of 25 studies were identified through the database search: six articles were selected and included. *Review articles, case report, descriptive articles, articles not focusing on test performance.

On 1st October 2024, a literature search in accordance with the preferred reporting items for systematic reviews and meta-analysis (PRISMA) guidelines was conducted using PubMed, web of sciences, and Scopus databases to identify relevant articles on free flap monitoring with implantable dopplers (see Figure 1, Figure 2).

The search algorithm used included two sets of keywords independently typed: “([{Cook Swartz} and {doppler}] and [flap]) and (monitoring)” and “([{Synovis} and {Flow Coupler}] and [flap]) and (monitoring).

The data were independently collected and analyzed by RB and AT.

The inclusion criteria were the following: clinical trials, case series, implantable dopplers, buried free flaps monitoring.

The exclusion criteria consisted of the following: previous reviews and metanalysis, case reports, expert descriptive articles, and animal studies.

Data on patients demographics, flap location/indication and devices performance in term of efficacy and effectiveness were collected. We particularly focused on the following indicators: sensitivity, specificity, positive predictive value (PPV), negative predictive value (NPV), false positive rate (FPR), false negative rate (FNR), failure rate, success rate and doppler placement (artery vs vein vs both) (See Table 1, Table 2, Table 3, Table 4 and Table 7, Table 8).

**Table 1:** Cook Swartz: characteristic of enrolled patient.

Author	Type	Number Of Patients/ Monitored Flaps	Sex	Age (Mean)	Preop-radiotherapy	Flap type	Flap Location	Buried Flaps (also) included	Indication for flap	Vein anastomosis (Coupler Vs Hand Sewn)	Doppler duration (Days)
Guillemaud et al., ^1^	R	351 / 384	244 men and 107 women	58.63	NR	Radial forearm (n = 261), fibula (n = 73), anterolateral thigh (n = 38), subscapular (n = 11), gracilis (n = 1)	H–N	NR	Tumor (n = 382); non tumor (n = 2)	Hand sewn	Mean: 8.95 days (Range 0–40)
Leibig et al.,^2^	R	129 / 147	83 men and 64 women	53.5 ± 18.1	26.5 %	Gracilis (n = 53); latissimus dorsi (n = 28); free vascularized scapular flap (n = 16); free fibular flap (n = 12); free iliac crest (n = 11); radialis flap (n = 26), ALT (n = 1)	H-N	NR	Tumor (n = 73); non tumor (n = 74: free muscle transfers for facial reanimation surgery; trauma; osteoradionecrosis; wound healing disorders)	Hand sewn	mean: 8
Paprottka et al.,^3^	R	35 / 35	15 men and 20 women	NR	NR	DIEP (n = 15), gracilis (n = 3), transverse muscolocutaneous gracilis (n = 1), ALT (n = 6); LD (n = 10)	H-N	yes ( %NR)	Tumor (n = NA), trauma (n = NA)	Hand sewn	mean: 10
Um et al.,^4^	R	76 /109	76 women	48.6	53.9 %	DIEP (n = 91), SIEA (n = 2), muscle sparing TRAM (n = 9); TUG (n = 7)	Breast	NR	Tumor (n = 109)	Coupler	mean: 5.2
Schmulder, et al.,^5^	R	473/226	244 men and 229 women	54.2	NR	NR	H-N, breast, limb	Yes	tumor (n = NA), trauma (n = NA), reanimation (n = NA), other (n = NA)	hand sewn	mean: 7
Hayler, et al.,^6^	P	99/100	74 men and 25 women	61.2 ± 12.1 (15.4–86.7)	NR	Radial forearm (n = 39), scapula tip (n = 20), ALT 8n = 15), fibula (n = 14), LD (n = 4), lateral border scapular (n = 4), parascapular (n = 2), infraclavicular (n = 1), gracilis (n = 1)	H-N	Yes	tumor (n = 100)	hand sewn	mean: 9.4 ± 2.3
Frost, et al.,^7^	P	20/21	9 men and 12 women	35–68 (range)	61.9 %	Jejunum (n = 2), DIEP (n = 9), rectus abdominis (1), TRAM (n = 1), fibula (n = 6), ALT (n = 1), LD (n = 1)	H-N, breast, limb, thoracic wall	Yes (n = 4)	Tumor (n = 18, 85.7 %), Non tumor (n = 3, 14.3 %)	hand sewn	range: 7–10
Chang, et al.,^8^	R	439/440	NR	54.5 ± 15.8	37.6 %	NR	H–N (n = 364), breast (n = 53), extremity free flaps (n = 22).	yes	NR	Hand sewn	NR
Lenz, et al.,^9^	R	69/69	52 men and 17 women	48	NR	Gracilis (n = 47), LD (n = 25), rectus abdominis (n = 37); vastus lateralis (n = 1) [skin grafted free muscle flaps + NPWT]	lower limb	no	Tumor (n = 6; 5 %); infection/chronic osteomyelitis (n = 14, 13 %), trauma (n = 55, 50 %), postoperative wound healing disorders (n = 30, 27 %), tissue defect due to impaired perfusion (n = 5, 5 %)	Hand sewn	At least 7 days (the dangle of the leg was usually started about 1 week after surgery and the Doppler signal was checked after every step)
Rozen, et al.,^10^	R	20 (20)	6 women, and 14 men	45.6 (15–73)	0 %	ALT (n = 2), parascapular (n = 1), LD (n = 12). lateral arm (n = 4), radial (n = 1)	lower limb	yes	Trauma/trauma sequelae (n = 20)	Hand sewn	28 days
Smit, et al.,^11^	R	103 (121) [145 Doppler probes]	103 women	50±9.5 (range: 22–67)	NR	DIEP (n = 102)), SIEP (n = 15), SGAP (n = 4)	breast	NR	Tumor (n = 121)	Hand sewn and coupler ( %NR)	7 days
Kimet al.,^12^	R	5 (5)	3 men	range: 57–66 years	NR	Chimeric thoracordorsal artery perforator free flap (n = 2), innervated chimeric latissimus dorsi muscle flap (n = ), chimeric latissimus dorsi and serratus anterior free flap (n = 1), free vascularised digastric lymph node transfer	H-N (n = 1), hand (n = 1),	yes	Tumor (n = 1), trauma (n = 1), facial paralysis (n = 1), recurrent ulcer (n = 1), lymphedema (n = 1)	Hand sewn	7 days
Whitaker et al.,^13^	R	103 (121)	103 women	NR	NR	DIEP (n = 106), SIEA (n = 15), SGAP (n = 4)	breast	no	Tumor (n = 121)	Hand sewn	NR
Oliver et al.,^14^	R	24 (24)	NR	NR	no	Gracilis, LD, TRAM, fibula, radial forearm, scapula, rectus	NR	no	NR	Hand sewn	7–14 days
Rozen et al.,^15^	R	8 (10)	8 women	NR	NR	DIEP (n = 6), SGAP (n = 4)	breast	yes	Tumor (n = 10)	Coupler (vein)	7 days
Pryor et al.,^16^	R	24 (24)	16 men and 8 women	57 (range: 14–78)	57 (14–78)	Radial forearm flap (n = 10), rectus abdominus (n = 6), fibula (n = 17), Anterolateral thigh (n = 13), scapula (n = 4)	H–N	yes (n = 6)	Tumor (n = 24)	Hand sewn	NR
Ho et al.,^17^	R	73 (75)	54 men and 19 women	59 (range: 17–84)	32.9 %	Fasciocutaneous radial forearm (n = 73), anterolateral thigfh (n = 17), iliac crest (n = 18) fibula (n = 13), scapula (n = 4), thoracordorsal system (n = 3), double flaps (radial forearm+iliac crest/fibula) (n = 2)	H–N	Yes (n = 10)	Tumor (n = 64), trauma (n = 1), osteoradionecrosis of mandible (n = 10)	Hand sewn	NR
Levine et al.,^18^	R	84 (134)	84 women	NR	NR	DIEP (n = 104), TUG (n = 10), IGAP (n = 2), PAP (n = 10), SGAP (n = 2), SIEA (n = 5), DFAP (n = 1)	Breast	Yes (n = 2) [total revision based in clinical monitoring: n = 78]	tumor (n = 134)	Coupler	3 days
Smit et al.,^19^	R	323 (323)	(NR) men and (NR) women	49.1 ±12.5 (range: 26–85)	yes ( % NR)	DIEP (n = NR), SGAP (n = NR), SIEA (n = NR), ALT (n = NR), radialis (n = NR), lateral arm (n = NR), LD (n = NR), fibula (n = NR), scapular (n = NR), gracilis (n = NR), serratus (n = NR)	Breast (n = 251), H–N (n = 39), extremities (n = 33)	NR	NR	Hand sewn	NR
Teven et al.,^20^	R	100 (100)	14 men and 86 women	51.8	47 %	VLNT (n = 100)	upper/lower limb, H-N	yes (n = 100)	Lymphedema	Hand sewn	7 days
Clert et al.,^21^	R	23 (23)	17 men and 6 women	58 (37–68)	yes ( % NR)	Fibula: n = 7; antebrachial: n = 15; scapular: n = 1	H-N	NR	Tumor (n = 20), osteoradionecrosis (n = 3)	hand sewn	14 days
Wax et al.,^22^	R	1142 (1142)	(NR) men and (NR) women	67 (3–97)	NR	Radial/ulna: n = 506; ALT: n = 117; rectus: n = 94; LD: n = 101; fibula: n = 187; radial osteocutaneous: n = 56; scapula: n = 25; jejunum: n = 39; enteral: n = 8; other: n = 9	H-N	NR	Tumor (n = 1142)	coupler	5 days
Horne et al.,^23^	R	119 (125)	NR	NR	No	Free gracilis: n = 125	H–N	NR	Long-standing facial paralysis	NR	NR
Rosenber et al.,^24^	R	20 (20)	NR	NR	NR	Fibula: n = 8; VRAM: n = 1; TRAM: n = 1; radial forearm: n = 1; ALT: n = 7; rectus abdominis: n = 1; jejunum: n = 1	Limb: n = 6; thorax: n = 1; H-N: n = 12; breast: n = 1	n = 20	Tumor (n = 20)	Hand sewn	NR
Park et al.,^25^	R	120 (120)	75 men nd 45 women	57	yes (25 %)	Scapula: n = 6; fibula: n = 54, radial forearm: n = 45, ALT: n = 17	H–N	NR	Tumor (n = 120)	Hand sewn	5 days
Fujiwara et al.,^26^	R	120 (120)	20 women, 80 men	62.9 (21–94)	22.5 %	Radial forearm (n = 60), fibula (n = 28), anterolateral thigh (n = 15), scapula (n = 7), LD (n = 6), other (rectus, scapula + LD) (n = 4)	H–N	0.8 %	Tumor (n = 79), other (n = 41)	Coupler	NR

Abbreviations: ALT, anterolateral thigh; DIEP, deep inferior epigastric perforator; HN, head and neck; LD, latissimus dorsi; NPWT, negative pressure wound therapy; NR , not reported; P, prospective; Preop, preoperative; R, retrospective; SGAP, superior gluteal artery perforator; TRAM, transverse rectus abdominus myocutaneous; TUG, transverse upper gracilis.

**Table 2:** Cook Swartz: indicators of effectiveness.

Author	Reduced/lost doppler signal [% on monitored flaps]	Salvage rate	False positive rates (FP/FP+TN)	False negative rate (FN/FN+TP)	Failure rate	Sensitivity-specificity-PPV-NPV	Placement
Guillemaud et al.,^1^	n = 46 (1″ %)	92.0 %	1.8 %	68.4 %	1.8 %	Sensitivity: 65.8 %;Specificity: 98.2 %; Positive predictive value: 80.7 %; Negative predictive value: 100 %	n = 318: A; n = 74: A ± V; n = 4: V
Leibig et al.,^2^	n = 29 (19.7 %)	63.0 %	1.7 %	37 %	6.8 %	sensitivity: 82.4 % (around the artery), 84.6 % (around the vein), (p=0.87); specificity: 89.2 % (around the artery), 96.1 % (around the vein), (p=0.17); PPV: 82.7 % (around the artery), 61.1 % (around the vein), (p=0.056); NPV: 96.7 % (around the artery), 94.2 % /around the vein), (p= 0.55)	n = 68: A; n = 79: V
Paprottka, et al.,^3^	n = 8 (22.9 %)	37.5 %	7.4 %	25 %	5.7 %	sensitivity 75 %, specificity 93 %, PPV 75 %, NPV 92.6 %	n = 35:VV
Um et al.,^4^	n = 11 (10.1 %)	81.8 %	1 %	0 %	1.8 %	sensitivity: 100 %; specificity: 99 %; PPV: 91.7 %; NPV: 100 %	n = 109: V
Schmulder, et al.,^5^	n = 36 (15.9 %)	95 %	1.6 %	0 %	12.1 %	sensitivity: 100 %; specificity: 98.4 %; PPV: 91.7 %; NPV: 100 %	n = 226: V
Hayleret al.,0^6^	n = 17 (12 %)	75.0 %	1 %	12 %	2 % [partial]	sensitivity: 87.1 %; specificity: 85.7 %; PPV: 98.8 %; NPV: 33.3 %	n = 100: A+V
Frostet al.,^7^	n = 3 (14.3 %)	33.3 %	0 %	33.3 %	66.7 % [partial and complete]	sensitivity: 66.7 %; specificity: 100 %; PPV: 100 %; NPV: 95 %	n = 3: A+V; n =13: V; n =5: A
Changet al.,^8^	n =56 (12.8 %)	62.5 %	11.6 %	22.2 %	4.8 %	sensitivity: 77.8 %; specificity: 88.4 %	n = 267: A; n =101: V; n = 71: A+V
Lenzet al.,^9^	n = 14 (20.3 %)	80 %	3.6 %	7.7 %	4.3 %	sensitivity: 92.3 %; specificity: 96.4 %; PPV:85.7 %; NPV: 98.2 %	n =69: V
Rozenet al.,^10^	n = 2 (10 %)	100 %	0 %	0 %	0 %	sensitivity: 100 %; specificity: 100 %; PPV:100 %; NPV: 100 %	n =20: V
Smitet al.,^11^	n = 15 (12.4 %)	86.7 %	6.7 %	0.9 %	1.7 %	sensitivity: 100 %; specificity: 99.2 %; PPV:93.3 %; NPV: 100 %	n = 145: V [in 51 flaps, a second venous anastomosis was done]
Kim et al.,^12^	n = 1 (20 %)	100 %	0 %	0 %	0 %	sensitivity: 100 %; specificity: 100 %; PPV: 100 %; NPV: 100 %	V
Whitaker et al.,^13^	n = 11 (9.1 %)	80 %	0.9 %	0 %	1.7 %	sensitivity: 100 %; specificity: 99.1 %; PPV:90.9 %; NPV: 100 %	n = 121: V
Oliver, et al.,^14^	n = 2 (8.3 %)	100 %	0 %	0 %	0 %	sensitivity: 100 %; specificity:100 %; PPV:100 %; NPV: 100 %	n = 24: V
Rozenet al.,^15^	N = 0	\	0 %	0 %	0 %	sensitivity: 100 %; specificity:100 %; PPV:100 %; NPV: 100 %	n = 10: V
Pryoret al.,^16^	N = 6 (25 %)	75 %	14.3 %	0 %	4 %	sensitivity; 100 %; specificity: 85.7 %; PPV: 50; NPV: 100 %	n = 12: A; n =11: V; n =1: A+V
Ho et al.,^17^	N = 9 (12 %)	62 %	4.5 %	33.3 %	6.7 %	sensitity: 66.7 %; specificity: 95.5 %; PPV: 66.7 %; NPV: 95.5 %	n = 67: A; n =7:V; n =1: A+V
Levine et al.,^18^	N = 2	0 %	0.8 %	50 %	2.2 %	sensitivity: 50 %; specificity: 99.2 %; PPV: 50 %; NPV= 99.2 %	n = 134: V
Smit, 2009^19^	n = 37 (10.4 %) (excluding one case of accidental remove of the device by the patient)	69 %	0.6 %	0 %	31 %	sensitivity: 100 %; specificity: 99.4 %; PPV: 71.4 %; NPV = 100 %	n = 323: V
Teven et al.,^20^	n = 5 (5 %)	100 %	2.1 %	0 %	0 %	sensitivity: 100 %; specificity: 97.7 %; PPV: 60 %; NPV = 100 %	n = 100: A
Clert et al.,^21^	n = 3 (13 %)	100 %	9.1 %	0 %	0 %	sensitivity: 100 %; specificity: 90.9 %; PPV: 33.3 %; NPV: 100 %	n = 20: V; n = 3: A
Wax et al.,^22^	n = 134 (11.7 %)	82 %	0.8 %	13 %	2.5 %	sensitivity: 87 %; specificity: 99 %; PPV: 89.3 %; NPV: 99.1 %	n = 43: V; n =1099: A
Horner et al.,^23^	NR	NR	NR	NR	NR	sensitivity: 100 %, specificity: 88 %; PPV: 41 %; NPV: 100 %	NR
Rosenberg et al.,^24^	n = 8 (40 %)	12.5 %	36.8 %	0 %	0 %	sensitivity: 100 %; specificity: 63.2 %; PPV: 12.5 %; NPV: 100 %	n = 12: V; n =6: A; n = 1: unspecified; n =1: flap perforator
Park et al.,^25^	n = 23 (19.2 %)	71.4 %	10.2 %	0 %	4.2 %	sensitivity: 100; specificity: 89.8 %; PPV: 52.2 %; NPV: 100 %	n = 120: A
Fujiwara et al.,^26^	n = 1 (0.8 %)	0 %	2.9 %	90.9 %	0.8 %	sensitivity: 9.1 %, spexificity: 97.1 %, PPV: 25 %, NPV: 90.9 %	n = 120: A

Abbreviations: A, artery; A+V, artery + vein; FN, false negative; FP, false positive; NPV , negative predictive value; PPV, positive predictive value; TN, true negative; TP, true positive; V = vein

**Table 3:** Synovis: characteristics of enrolled patients.

Author	type of study	Patients/flap	Flow coupler anastomosis	Sex	Age (mean)	Pre-operative radiotherapy	Flap type	Recipient vessel	Flap location	Buried flaps (also) included	Flap indication	Synovis duration (days)
Um, 2014^4^	R	74 (111)	NR	74 women	50.3	47.3 %	DIEP (n = 90), SIEA (n =6), MS TRAM (n =10), TUG (n =3), SGAP (n =2)	NR	breast	NR	tumor (n =74)	mean: 4.8 days
Creasy, 2023^27^	R	106 (126)	185 (NR)	106 women	NR	NR	DIEP (n =45), PAP (n =21), ALT (n =41), TFL (n =2), VL (n =1), TDAP (n =2), LD (n =1), RFFF (n =4), SCIP (n =1), MFC periosteal (n =6)	NR	NR	yes ( %NR)	tumor(n =106)	3–5 days
Shtarbanov, 2024^28^	R	16 (18)	19 (n =3: 2 mm; n =13: 2.5 mm; n =2: 1.5 mm; n =1: 3 mm)	NR	NR	NR	NR	NR	hand, breast, lower limb	yes ( %NR)	tumor (n =16)	NR
Zhang, 2012^29^	R	18(19)	20 (n = 5: 2 mm; n =8: 3 mm; n =7: 2.5 mm)	11 women and 7 men	20–76	NR	LD (n =2), fibula (n =7), radial forearm (n =2), anterolateral thigh (n =4), DCIA (n =2), scapula (n =1), lateral arm (n =1)	cefhalic (n =1), external jugular (n =6), internal jugular (n =1), anterior jugular (n =1), facial (n =9), retromandibular (n =1), superficial temporal (n =1)	head and neck	yes ( %10.5 %)	tumor (n =11), ostreomyelitis (n =2), osteoradionecrosis (n =2), other (n =3)	5–6 days
Chadwig, 2019^30^	R	14(26)	24 (n =12: 2.5 mm; n = 8: 3 mm; n =2: 3.5 mm)	14 women	39 (24–58)	NR	DIEP (n =26)	IMV	breast	yes (100 %)	tumor (n =7), risk reducing mastectomy (n =7)	4.5 days
Fujiwara, 2018^26^	R	120 (120)	191 (2.5 mm, range, 1.5–4.0)	20 women, 80 men	62.9 (21–94)	22.5 %	radial forearm (n =60), fibula (n =28), anterolateral thigh (n =15), scapula (n=7), LD (n=6), other (rectus, scapula+LD) (n=4)	external jugular (n=85), common facial (n=53), branch of common facial (n=16), facial (n=16), superior thyroid (n=10), internal jugular (n=10), anterior jugular (n=6), other (n=3)	H-N	yes (NR %)	tumor (n=79), other (n=41)	NR

Abbreviations: ALT, anterolateral thigh; DCIA, deep circumflex iliac artery; DIEP, deep inferior epigastric perforator; HN, head and neck; LD, latissimus dorsi; MFC, medial femoral condyle; MS- TRAM, muscle sparing transverse rectus abdominus myocutaneous; NPWT = negative pressure wound therapy; NR, not reported; P, prospective; PAP, profunda artery perforator; Preop, preoperative; R, retrospective; RFFF, radial forearm free flap; SCIP, superficial circumflex iliac perforator; SGAP, superior gluteal artery perforator; TFL, tensor fascia latae; TDAP, thoracodorsal artery perforator; TRAM, transverse rectus abdominus myocutaneous; TUG, transverse upper gracilis; VL, vastus lateralis;

**Table 4:** Synovis: indicators of effectiveness.

Author	Reduced/Lost Doppler Signal [ % On Monitored Flaps]	Salvage Rate	False Positive Rates (FP/FP+TN)	False Negative Rate (FN/FN+TP)	Failure Rate	Sensitivity-Specificity-PPV-NPV	Anastomosis	Complications Related To The Device
Um, 2014^4^	4.5 %	50 %	1.9 %	0 %	0.9 %	sensitivity: 100 %, specificity: 98.1 %, PPV: 66.7 %, NPV: 100 %	E-E	0.9 % vein kinking caused by the device
Creasy, 2023^27^	2.4 %	100 %	0 %	0 %	0 %	NR	NR	no
Shtarbanov, 2024^28^	31.3 %	100 %	20 %	0 %	5.5 %	sensitivity: 100 %, specificity: 80 %, PPV: 60 %; NPV: 100 %	E-E	16.7 % vein twisting caused by the device
Zhang, 2012^29^	30 % [due to: n=2: inadvertent intraoperative removal; n=2: vessel kinking; n=2: equipment failure]	100 %	0 %	0 %	0 %	sensitivity: 100 %, specificity: 100 %, PPV: 100 %; NPV: 100 %	NR	10.5 % vein twisting caused by the device
Chadwig, 2019^30^	4.2 %	100 %	4.2 %	0 %	0 %	sensitivity: 100 %, specificity: 95.8 %, PPV: 0 %; NPV: 100 %	NR	no
Fujiwara, 2018^26^	8.3 %	90 %	13.6 %	0 %	0.8 %	sensitivity:100 %, specificity: 86.4 %; PPV: 44 %; NPV: 100 %	NR	no

Abbreviations: E-E = end to end; FN = false negative; FP = false positive; NPV = negative predictive value; PPV = positive predictive value; TN = true negative; TP = true positive; V = vein

Data were recorded and tabulated using Microsoft Excel (Microsoft Corp., Redmond, Washington, Version 2210).

## Quality assessment

The risk of bias of each study is reported in Supplementary Table 1.

## Results

From January 2019 to February 2024, 179 free flaps (see table 1) were operated on at our institution. Of these, 52 were monitored using implantable dopplers of which 39 were Synovis flow couplers (Synovis Life Technologies, Inc., St. Paul, MN) and 14 Cook Swartz (Cook Medical, Bloomington, IN). Of the 39 Flow Couplers, 29 were used in breast and nine in head and neck surgery, whereas of the 14 Cook Swartz, two were used in breast surgery and 12 in head and neck surgery. All the monitored flaps were buried. With the flow couplers, we had one case of false positivity and one case of flap failure, both in the head and neck group. These entailed a specificity of 97.2 % and a PPV of 50 %, with 100 % sensitivity and NPV. The false-positive case corresponded to a flap where the signal was barely detectable, yet clinically the flap appeared to be viable; conversely, the failed case involved a condylar flap that had already experienced complications intraoperatively. The Cook Swartz performed better, with only one case of flap failure, in a true positive labelled patient undergone head and neck surgery.

To understand if we were getting the most out of these devices, the literature was systematically explored. The search using Cook Swartz thesaurus identified 116 articles while that of Synovis Flow Coupler 25 articles. After removing duplicates (n = 41 and n = 10, respectively) and articles that did not meet the inclusion criteria (n = 51 and n =7, respectively), we collected 26 and six articles, respectively (see Tables 1,2,3,4).

Table 7, Table 8 show a summary of the indicators of effectiveness of the two devices according to the literature.

Our results (Table 5, Table 6) are in line with those reported in literature, with a worse performance in terms of specificity and PPV of Synovis as compared to Cook Swartz.

**Table 5:** Our casistics.

	Conventional anastomosis	Implantable Doppler	Flap failure
n = 179	n = 127 (70.9 %)	n = 52 (29.1 %)	n = 2 (1.1 %)
Head and neck: n = 120 (67 %) Breast: n = 46 (25.7 %) Upper limb: n = 10 (5.6 %) Lower limb: n = 3 (1.7 %)

**Table 6:** Our results.

N = 38 flow couplers (n=29 breast; n=9 head and neck)	N =14 Cook Swartz (n=2 breasts, n=12 head and neck)
N =36 true negatives N =1 true positive (failed) (head and neck) N = 1 false positive (head and neck) N = 0 false negative	N = 13 true negatives N = 1 true positive (failed) (head and neck) n = 0 false positive n = 0 false negative
Sensitivity: 100 %	Sensitivity: 100 %
Specificity: 97.1 %	Specificity: 100 %
PPV: 50 %	PPV: 100 %
NPV: 100 %	NPV

Abbreviations: NPV, negative predictive value; PPV, positive predictive value

**Table 7:** Cook Swartz: summary of the pertinent literature.

Cook Swartz	
26 articles	
4102 patients e 4038 flaps	
Time to Doppler removal	3–28 days
Flap revision according to changed CS signal:	0 %-40 %
Salvage rate	0–100 %
Failure rate	0- 66.7 %
FPR	0–36.8 % (81 % of studies ≤ 10 % and *54 % ≤ 2 %)*
FNR	0 %-90.9 % (*46 % of studies ≤ 10 %* and 23 % > 30 %)
Sensitivity	9–100 %; *> 90 % in 62 % of studies* (up to 100 % in 58 %)
Specificity	63–100 %; *> 90 % in 73 % of studies*
**PPV**	12.5 % -100 %; >90 % in 38.5 % of studies
**NPV**	33.3 % -100 %; > 90 % nel 96.2 %

**Table 8:** Synovis: summary of the Pertinenti literature.

Synovis	
7 articles	
348 patients and 420 flaps	
Time to Synovis removal:	4.8–6 days
Flap revision according to changed FC signal	2.4–30 %
Salvage rate	50–100 %
Failure rate	0- 5.5 %
FPR	*0–20 %*
FNR	0 %
Sensitivity	100 %
Specificity	*80–100 %*
PPV	0–100 %
NPV	**100 %**

## Discussion

For fasciocutaneous flaps transferred to body regions with easy observation, the only monitoring required is clinical.^6^^,^^8^ Buried flaps, muscle flaps without monitor islands, skin grafted muscle flaps, quite the opposite, can only be monitored accurately with the use of implantable probes or exposed paddles for clinical assessment.^6^^,^^8^ Implantable probes also allow for the postoperative concept of negative pressure wound therapy (NPWT) of free muscle flaps.^8^^,^^9^

In our daily practice, the criterion upon which we base the decision to use an implantable Doppler is whether the flap is buried. This is the rationale behind the use of Doppler monitoring in the head and neck as well as breast reconstructions, where flaps are typically buried. In head and neck reconstructions, for example, nearly all flaps are buried or semi-buried, and the use of a non-clinical monitoring system becomes necessary due to the frequent intraoral location of the flap, which is subject to color and turgor changes caused by saliva (maceration tends to give the flap a pale appearance), as well as the difficulty in having the patient open their mouth due to spasms in the postoperative period. In contrast, for free flaps monitoring in the extremities (lower/upper limbs), such an expensive system is not recommended, as the flaps are usually not buried and clinical monitoring remains the gold standard. In clinically monitorable flaps, the use of the probes may be beneficial only if it also facilitates faster performance, as is the case with the Synovis device, which functions as a coupler and releases the probe simultaneously with the suturing process (being a coupler it also helps to ensure mechanical patency). Conversely, the Cook-Swartz system consists solely of a probe that is positioned on the anastomosis after it has been completed.

The main flaws of clinical monitoring are its dependence on clinical experience and the fact that when a vascular problem becomes clinically evident, often the flap is irreparably damaged.^13^ In most cases, the ability to recognize a compromised flap and subsequent early re-exploration is the key to successful salvage, as most successful salvages occur within the first 24 hours.^6^

The implantable Doppler probes allow the direct measurement of blood flow since a monitor is placed onto the pedicle thus helping detect flap compromise before clinical ischemia becomes evident.^3^ The early detection of flap compromise and prompt return to the operating room can influence free flap salvage rates by returning perfusion to the flap before the no-reflow phenomenon occurs.^4^^,^^5^ In the current literature, the rate of salvage varies from 33 % to 89 %.^4^

The two devices allowing a continuous monitoring of flow across the anastomosis are the Cook Swartz and the Synovis Flow Coupler. These implantable probes have the strength of continuous flow evaluation of the pedicle flow opposite to the other monitoring methods (microdyalisis, clinical assessment, near-infrared spectroscopy, microdialysis, laser Doppler flowmetry combined with tissue spectrophotometry or modified oxygen microelectrode …) which only provide indirect evidence of impaired pedicle blood flow by measuring capillary perfusion and therefore producing critical latency.^6^^,^^8^ No Difference (p > 0.05) between the two devices in terms of their accuracy in detecting vessel compromise have been identified in the clinical setting.^4^ The singular advantage of the Flow Coupler is that it allows the Doppler to be placed simultaneously at the time of anastomosis, eliminating the need for any additional intraoperative procedures.^4^

Different studies of the Cook Swartz implantable Doppler showed a significant increase in flap salvage rates contrary to clinical assessment (96.14 % vs. 89.27 %)^5^^,^^20^ without increasing the rate of false-positive operative re-explorations and concluded that it could be used as a stand-alone measure of flap status.^4^, ^5^, ^6^ In buried flaps, the difference in flap salvage between implantable Dopplers and clinical exam is even larger (94 % vs 40 %) because buried flaps cannot be evaluated clinically and it is difficult to confirm that they are healthy without operative exploration. The risk of an increased false positive rate is not a serious drawback for free flaps that are not buried, because false-positive signals can be confirmed as falsely positive by clinical examination and ignored. However, for completely buried free flaps, there is no accepted rapid and reliable confirmatory test, so one must resort to operative exploration, which is an invasive procedure that is both time consuming and labor intensive.^24^

The risk of false negatives has been reported for the Cook Swartz venous placement (an arterial signal could persist even in the setting of a venous thrombosis)^31^ and for both the devices in head and neck flaps where there exists the problem of transmitted vessel interference, due to the proximity of the large-calibre vessels, namely the carotid artery and the internal jugular vein leading to FN. In the neck, venous signals are also prone to false positive findings, because of their variation according to the head position and to the respiratory cycle. When the venous signal appears to be missed, two dynamic manoeuvers have been described to increase the venous flow and the signal perception reducing the false positive rate: the “whoosh test” (which consist of exerting a digital pressure over the flap) and the “heave test” (a Valsalva Manoeuver).^21^^,^^22^

Our early experience, combined with literature findings, has progressively prompted us to prefer the use of the Flow Coupler in buried diep flap (where it also aids in the anastomosis making) while reducing its application in buried head and neck flaps so as to reduce the false positive rates and the subsequent unnecessary operating room take back. In DIEP flaps, the most critical point is the venous anastomosis, which is technically demanding, as the mammary artery exhibits significant pressure and is less likely to undergo occlusion. That is why, in DIEP flaps, we tend to place the probe on the vein. Conversely, in head and neck reconstructions, where venous pressure is lower (due to variations corresponding to the respiratory cycle, etc.), we routinely perform two venous anastomoses and one arterial anastomosis. In this scenario, occlusion of both veins is less likely, making the artery the critical point to monitor. Therefore, in head and neck cases, we typically position the doppler (Cook Swartz) probe on the artery.

In the head and neck surgery, our current trend is to use the Cook Swartz placed around the arteries, being these less prone to positional changes and respiratory cycle oscillations.

An important advantage of implantable dopplers as direct monitoring techniques versus the clinical monitoring is also observed during the intraoperative period, due to disclosure of any kind of problem (blood disturbance and pressure on the vascular pedicle), from the moment, the implantable Doppler was connected until patient education about appropriate neck positions and inappropriate movements based on the signal shown on the monitor's screen.^5^^,^^11^

The system is based on a “yes” versus “no” response (audible signal vs signal absence) and does not require any interpretation or medical education. The patients themselves can alert the medical staff if the signal shades away. Furthermore the presence of a constant sound reassures and relieves anxiety on patients who can personally monitor their flap status. Among the limits of the present study we have to cite its retrospective design, the small cohort of patients included and the fact that the patients were not randomized to one device or another but the first were monetarized using Synovis and the last by Cook Swartz after the first case of false positivity. Furthermore data span over a period of five years, possibly reflecting a learning curve in our ability to use them. Another important drawback of this study is the lack of a statistical analysis between the performances of the two devices. Thus our results, although in line with the current literature, must be read with caution.

Future studies should focus on AI-assisted Doppler interpretation and cost-analysis comparisons between the two systems in different body parts.

## Conclusion

Implantable dopplers are a valid supplement to clinical monitoring which cannot be considered the gold standard for all free flaps. Buried flaps, skin grafted muscle flaps, intra-oral flaps, flaps in dark-skin patients are better monitored with implantable dopplers, which also ensure a continuous check of pedicle flow, thus revealing vascular problems in advance (before the no reflow phenomenon occurs) also in fasciocutaneous flaps. To reduce the risk of false positive results (which in buried flap cannot be excluded without theatre take back) we use the Cook Swartz doppler probe around the artery in the head and neck flaps, while we use the Synovis flow coupler in diep flaps where the anatomical location of recipient vessel is less prone to variations of the flow.

## Funding

None.

## Declaration of competing interest

The authors have no conflict of interest to declare.

## Appendix Supplementary materials

Image, application 1
