# Supplementary material for: Free flaps monitoring using implantable doppler: our experience and of the literature
Source: JPRAS Open. 2025 Sep 5;46:427–45. doi: 10.1016/j.jpra.2025.08.023 (PMC12605636; doi:10.1016/j.jpra.2025.08.023)
Supplement: Supplementary file 1 [file mmc1.docx]

The risk of bias of each study included in the review was evaluated using the checklist for observational cohorts and cross-sectional studies reported by the National Institutes of Health26 which consists of 14 items that explore the intrinsic validity of the study and the related risk of bias. The final quality rating consist of a qualitative judgement (“good”, “fair”, or “poor”) that the review authors are asked to make having 14 questions given as a guide (https://www.nhlbi.nih.gov/health-topics/study-quality-assessment-tools).

1. Was the research question or objective in this paper clearly stated?

2. Was the study population clearly specified and defined?

3. Was the participation rate of eligible persons at least 50%?

4. Were all the subjects selected or recruited from the same or similar populations (including the same time period)? Were inclusion and exclusion criteria for being in the study prespecified and applied uniformly to all participants?

5. Was a sample size justification, power description, or variance and effect estimates provided?

6. For the analyses in this paper, were the exposure(s) of interest measured prior to the outcome(s) being measured?

7. Was the timeframe sufficient so that one could reasonably expect to see an association between exposure and outcome if it existed?

8. For exposures that can vary in amount or level, did the study examine different levels of the exposure as related to the outcome (e.g., categories of exposure, or exposure measured as continuous variable)?

9. Were the exposure measures (independent variables) clearly defined, valid, reliable, and implemented consistently across all study participants?

10. Was the exposure(s) assessed more than once over time?

11. Were the outcome measures (dependent variables) clearly defined, valid, reliable, and implemented consistently across all study participants?

12. Were the outcome assessors blinded to the exposure status of participants?

13. Was loss to follow-up after baseline 20% or less?

14. Were key potential confounding variables measured and adjusted statistically for their impact on the relationship between exposure(s) and outcome(s)

| author | Qs.1 | Qs.2 | Qs.3 | Qs.4 | Qs.5 | Qs.6 | Qs.7 | Qs.8 | Qs.9 | Qs.10 | Qs.11 | Qs.12 | Qs.13 | Qs.14 | Final rating |
| --- | --- | --- | --- | --- | --- | --- | --- | --- | --- | --- | --- | --- | --- | --- | --- |
| Chadwig, 2019 | Yes | Yes | Yes | Yes | Yes | Yes | Yes | NA | Yes | NA | Yes | NA | Yes | Yes | GOOD |
| Chang, 2016 ^8^ | Yes | Yes | Yes | Yes | Yes | Yes | Yes | NA | Yes | NA | Yes | NA | Yes | Yes | GOOD |
| Clert, 2013 ^21^ | Yes | Yes | Yes | Yes | Yes | Yes | Yes | NA | Yes | NA | Yes | NA | Yes | Yes | GOOD |
| Creasy, 2023 | Yes | Yes | Yes | Yes | Yes | Yes | Yes | NA | Yes | NA | Yes | NA | Yes | Yes | GOOD |
| Frost, 2014 ^7^ | Yes | Yes | Yes | Yes | Yes | Yes | Yes | NA | Yes | NA | Yes | NA | Yes | Yes | GOOD |
| Fujiwara, 2018 ^26^ | Yes | Yes | Yes | Yes | Yes | Yes | Yes | NA | Yes | NA | Yes | NA | Yes | Yes | GOOD |
| Guillemaud, 2008 ^1^ | Yes | Yes | Yes | Yes | Yes | Yes | Yes | NA | Yes | NA | Yes | NA | Yes | Yes | GOOD |
| Hayler, 2020 ^6^ | Yes | Yes | Yes | Yes | Yes | Yes | Yes | NA | Yes | NA | Yes | NA | Yes | Yes | GOOD |
| Ho, 2014 ^17^ | Yes | Yes | Yes | Yes | Yes | Yes | Yes | NA | Yes | NA | Yes | NA | Yes | Yes | GOOD |
| Horner, 2023 ^23^ | Yes | Yes | Yes | Yes | Yes | Yes | Yes | NA | Yes | NA | Yes | NA | Yes | Yes | GOOD |
| Kim, 2013 ^12^ | Yes | Yes | Yes | Yes | Yes | Yes | Yes | NA | Yes | NA | Yes | NA | Yes | Yes | GOOD |
| Leibig, 2019 ^2^ | Yes | Yes | Yes | Yes | Yes | Yes | Yes | NA | Yes | NA | Yes | NA | Yes | Yes | GOOD |
| Lenz, 2017 ^9^ | Yes | Yes | Yes | Yes | Yes | Yes | Yes | NA | Yes | NA | Yes | NA | Yes | Yes | GOOD |
| Levine, 2013 ^18^ | Yes | Yes | Yes | Yes | Yes | Yes | Yes | NA | Yes | NA | Yes | NA | Yes | Yes | GOOD |
| Oliver, 2005 ^14^ | Yes | Yes | Yes | Yes | Yes | Yes | Yes | NA | Yes | NA | Yes | NA | Yes | Yes | GOOD |
| Paprottka, 2020 ^3^ | Yes | Yes | Yes | Yes | Yes | Yes | Yes | NA | Yes | NA | Yes | NA | Yes | Yes | GOOD |
| Park, 2018 ^25^ | Yes | Yes | Yes | Yes | Yes | Yes | Yes | NA | Yes | NA | Yes | NA | Yes | Yes | GOOD |
| Pryor, 2006 ^16^ | Yes | Yes | Yes | Yes | Yes | Yes | Yes | NA | Yes | NA | Yes | NA | Yes | Yes | GOOD |
| Rosenberg, 2006 ^24^ | Yes | Yes | Yes | Yes | Yes | Yes | Yes | NA | Yes | NA | Yes | NA | Yes | Yes | GOOD |
| Rozen, 2009 ^15^ | Yes | Yes | Yes | Yes | Yes | Yes | Yes | NA | Yes | NA | Yes | NA | Yes | Yes | GOOD |
| Rozen, 2010 ^10^ | Yes | Yes | Yes | Yes | Yes | Yes | Yes | NA | Yes | NA | Yes | NA | Yes | Yes | GOOD |
| Schmulder, 2011 ^5^ | Yes | Yes | Yes | Yes | Yes | Yes | Yes | NA | Yes | NA | Yes | NA | Yes | Yes | GOOD |
| Shtarbanov, 2024 | Yes | Yes | Yes | Yes | Yes | Yes | Yes | NA | Yes | NA | Yes | NA | Yes | Yes | GOOD |
| Smit, 2008 ^11^ | Yes | Yes | Yes | Yes | Yes | Yes | Yes | NA | Yes | NA | Yes | NA | Yes | Yes | GOOD |
| Smit, 2009 ^19^ | Yes | Yes | Yes | Yes | Yes | Yes | Yes | NA | Yes | NA | Yes | NA | Yes | Yes | GOOD |
| Teven, 2017 ^20^ | Yes | Yes | Yes | Yes | Yes | Yes | Yes | NA | Yes | NA | Yes | NA | Yes | Yes | GOOD |
| Um, 2014 ^4^ | Yes | Yes | Yes | Yes | Yes | Yes | Yes | NA | Yes | NA | Yes | NA | Yes | Yes | GOOD |
| Wax, 2013 ^22^ | Yes | Yes | Yes | Yes | Yes | Yes | Yes | NA | Yes | NA | Yes | NA | Yes | Yes | GOOD |
| Whitaker, 2010 ^13^ | Yes | Yes | Yes | Yes | Yes | Yes | Yes | NA | Yes | NA | Yes | NA | Yes | Yes | GOOD |
| Zhang, 2012 | Yes | Yes | Yes | Yes | Yes | Yes | Yes | NA | Yes | NA | Yes | NA | Yes | Yes | GOOD |

Table 5 – Risk of bias of included studies valuated using the checklist for observational cohorts and cross-sectional studies reported by the National Institutes of Health (<https://www.nhlbi.nih.gov/health-topics/study-quality-assessment-tools>)

Abbreviations:

NA = not applicable; Qs. = question
